# Evolving regulatory perspectives on digital health technologies for medicinal product development

**DOI:** 10.1038/s41746-023-00790-2

**Published:** 2023-03-29

**Authors:** Seya Colloud, Thomas Metcalfe, Scott Askin, Shibeshih Belachew, Johannes Ammann, Ernst Bos, Timothy Kilchenmann, Paul Strijbos, Damien Eggenspieler, Laurent Servais, Chloé Garay, Athanasios Konstantakopoulos, Armin Ritzhaupt, Thorsten Vetter, Claudia Vincenzi, Francesca Cerreta

**Affiliations:** 1 F. Hoffmann-La Roche Ltd., Basel, Switzerland; 2 Novartis Pharma AG, Basel, Switzerland; 3 Biogen Digital Health International GmbH, Baar, Switzerland; 4 SYSNAV, Vernon, France; 5 Muscular Dystrophy UK Oxford Neuromuscular Centre, Department of Paediatrics, University of Oxford, Oxford, UK; 6 Division of Child Neurology, Centre de Références des Maladies Neuromusculaires, Department of Paediatrics, University Hospital Liège and University of Liège, Liège, Belgium; 7 Eli Lilly and Company Ltd., Basingstoke, UK; 8 Bristol Myers Squibb, Uxbridge, UK; 9 European Medicines Agency, Amsterdam, The Netherlands; 10 Present Address: GE Healthcare S.A., Athens, Greece

**Keywords:** Medical research, Biomarkers, Scientific community, Health care

## Abstract

Digital health technology tools (DHTTs) present real opportunities for accelerating innovation, improving patient care, reducing clinical trial duration and minimising risk in medicines development. This review is comprised of four case studies of DHTTs used throughout the lifecycle of medicinal products, starting from their development. These cases illustrate how the regulatory requirements of DHTTs used in medicines development are based on two European regulatory frameworks (medical device and the medicinal product regulations) and highlight the need for increased collaboration between various stakeholders, including regulators (medicines regulators and device bodies), pharmaceutical sponsors, manufacturers of devices and software, and academia. As illustrated in the examples, the complexity of the interactions is further increased by unique challenges related to DHTTs. These case studies are the main examples of DHTTs with a regulatory assessment thus far, providing an insight into the applicable current regulatory approach; they were selected by a group of authors, including regulatory specialists from pharmaceutical sponsors, technology experts, academic researchers and employees of the European Medicines Agency. For each case study, the challenges faced by sponsors and proposed potential solutions are discussed, and the benefit of a structured interaction among the different stakeholders is also highlighted.

## Introduction

Digital health technology tools (DHTTs) for use in conjunction with medicinal products are being developed to empower patients to better manage their own treatment^1^ and have the potential to transform clinical trials^2–4^. Thanks to DHTTs, clinical trial endpoints can be measured in a home setting and may provide higher external validity and sensitivity in detecting medicinal products’ efficacy^3^. This, in turn, could lead to leaner clinical trials, reducing the burden on patients^2^ and bringing medicines to patients faster.

**Fig. 1:**
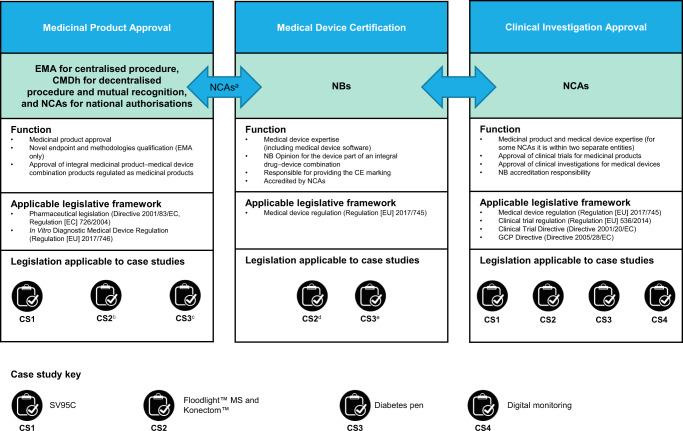
Summary of regulatory bodies, their function, and the legislative framework relevant to digital health technology tools. Summary of the regulatory bodies responsible for medicinal product approval, medicine device certification and clinical investigation approval. Arrows represent formal interaction between regulatory bodies. Listed are the relevant legislative frameworks for each regulatory body and indicated below are the case studies where these frameworks are applicable. ^a^For Article 117, interaction with NBs occurs via the manufacturer. ^b^For the clinical trial tool. ^c^For the injector pen. ^d^For the SaMD functionality in clinical practice. ^e^For the smartphone app. *CE* Conformitè Europëenne, *CMDh* Co-ordination Group for Mutual Recognition and Decentralised Procedures–human, *CS* case study, *EC* European Council, *EMA* European Medicines Agency, *EU* European Union, *GCP* Good Clinical Practice, *MDSW* medical device software, *MS* multiple sclerosis, *NB* Notified Body, *NCA* National Competent Authority, *SV95C* Stride Velocity 95th Centile.

As DHTTs are being used to collect data and substantiate the safety and efficacy of medicinal products in clinical trials for marketing authorisation applications (MAAs), medicines regulators (European Medicines Agency [EMA] and European Union [EU] national competent authorities [NCAs]) must ensure that the clinical evidence generated is representative, robust and scientifically valid. DHTTs that also meet the definition of a medical device are subject to medical device regulatory oversight by the relevant device body (NCAs with device competence and Notified Bodies [NBs]); both device bodies also play a role in the risk classification of medical devices^5^. For this reason, DHTT development in the EU takes place at the intersection of the medical device and the medicinal product regulatory frameworks, making it a challenging environment for sponsors of DHTTs and medicine developers to navigate. A high-level summary of the different regulatory authorities and their function is outlined in Fig. 1. The EMA reviews whether the data generated by DHTTs are appropriate to support the benefit–risk assessment of medicinal products subject to the centralised procedure. This can take place either during the assessment of the MAA of a medicinal product during which the DHTT-derived evidence has been submitted, or preferably earlier in the context of the DHTT development using the EMA Qualification of Novel Methodologies (QoNM) platform^6^.

**Fig. 2:**
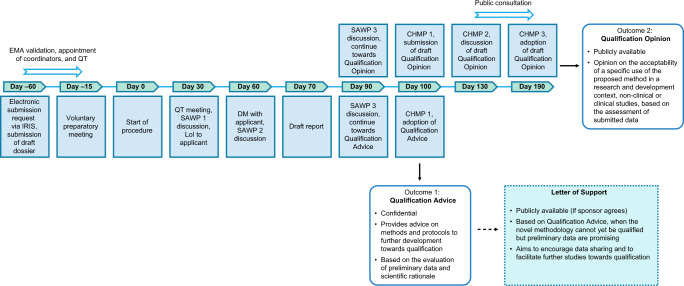
Summary of the EMA qualification procedure for digital technology-based methodologies to support approval of medicinal products. Procedure summary for the qualification for digital technology-based methodologies to support the approval of medicinal products, including the potential application outcomes. *CHMP* Committee for Medicinal Products for Human Use, *DM* discussion meeting, *EMA* European Medicines Agency, *LoI* List of Issues, *QT* qualification team, *SAWP* scientific advice working party.

The EMA qualification process assesses whether data derived by a DHTT are acceptable to support a MAA (or MAA variation or extension) and whether the methodology allows for a valid and clinically meaningful interpretation of the concept of interest in a reliable and robust manner. Applicants may request Qualification Advice at any time during the development process to ensure appropriate method development^7^. An iterative qualification process is possible and desirable to allow refinement of validation plans as knowledge progresses. The qualification procedure can have various outcomes, outlined in Fig. 2. A satisfactory qualification procedure leads to publication of a Qualification Opinion^7^. If the novel methodology cannot yet be qualified, the sponsor will receive confidential Qualification Advice and publication of a Letter of Support may be offered if preliminary data are considered promising. Publication of a Qualification Opinion attests the adequacy of a methodology for its context of use to generate data for medicinal product benefit–risk assessment^7^.

It is possible to obtain scientific advice on medicinal products both from NCAs and the Committee for Medicinal Products for Human Use (CHMP); for medical devices, scientific advice can be sought from NCAs with competency on medical devices. NCAs, with competency on medicinal products and medical devices, can also be invited to attend Innovation Task Force (ITF) meetings^8^, which provide a forum for early dialogue with applicants on innovative aspects in medicines development including emerging therapies and technologies. To date, NBs are not allowed to give pre-certification services, e.g., providing advice, before an application is lodged by the manufacturer, and therefore these services have to take place under the scope of the application.

In its ‘Regulatory Science Strategy to 2025’ (RSS), the EMA envisages the creation of an integrated evaluation pathway for the assessment of medical devices, in vitro diagnostics and borderline products^9^. Among the goals of the integrated pathway assessment, three of them are relevant also for DHTTs: 1. to establish a process for multi-stakeholder scientific advice to support development of medicine–device combinations, qualification methodologies and the use of companion diagnostics; 2. to create a process to consult medical device authorities and/or NBs (as applicable) for device-related aspects throughout the product lifecycle, including post-authorisation safety-related events; 3. to adapt consultation processes to address digital technologies and wearables^9^.

The four case studies presented illustrate the complexity of the different regulatory frameworks that span across DHTTs used in medicines development and highlight the benefit of increased collaboration between various stakeholders including device and medicine regulators and cross-industry collaboration. A group, including regulatory specialists from pharmaceutical sponsors, technology experts, academic researchers and employees of the EMA, collected and analysed the cases. Some members of this group have previously published on the use of digital technologies in medicines development^10^ and supported the development of the EMA’s Q&A^11^ on this topic.

## Case studies

The four case studies presented include DHTTs at various stages of medicines development across a variety of disease areas including multiple sclerosis (MS), diabetes and cancer. Some of the DHTTs analysed may be classified as medical devices, depending on their intended purpose in clinical practice. Case studies 1–2 are examples where sponsors are leveraging the QoNM for Medicines Development to obtain regulatory endorsement of their DHTT methodologies, independent of a particular medicinal product. Case study 3 is an example where the potential impact of DHTTs on the benefit–risk assessment of the medicinal product may also be discussed as part of scientific advice and later assessed at the time of MAA or post-approval. Case study 4 is an example where the DHTT may only be subject to medical device regulatory oversight in Europe. For each case, the industry authors have highlighted the core challenges faced in navigating the regulatory frameworks and potential solutions have been explored, with the aim of further supporting the development and use of DHTTs.

## Case study 1—Qualification of a digital endpoint for measurement of real-world ambulation in Duchenne muscular dystrophy (Stride Velocity 95th Centile)

### What is the technology/how is it used?

Stride Velocity 95th Centile (SV95C) is a digital clinical outcome assessment (COA) and is the first digital endpoint, collected by a wearable device, to have received endorsement by the EMA through publication of a Qualification Opinion^12,13^. A wearable medical device based on magneto-inertial technology (ActiMyo^®^) was used to measure patient movements in a non-controlled environment^14,15^. The device is passive, meaning it does not require patients to complete any tasks during their daily lives^13^, and has been through extensive validation both in controlled and uncontrolled environments with control and patient populations. Algorithms transform the data from the device into physical variables, such as stride length and speed, and then compute clinical variables, such as the SV95C, from the physical variables^13^. The EMA qualification validates that the SV95C is accurate, reliable, sensitive to change and is relevant to patients. More specifically, the SV95C that quantifies a patient’s maximal ambulation velocity in a continuous manner in a home environment and reflects most components (except for additional information like endurance, or confounders like motivation or fatigue at time of assessment) of the well-established Six-Minute Walking Test (6MWT)^13^.

### Regulatory aspects

The ActiMyo wearable device, together with the SV95C digital endpoint, is a DHTT that spans across the medical device and the medicinal product regulatory frameworks. The Medical Device Directive (now updated to the Medical Device Regulation [MDR] 2017/745), the Good Clinical Practice Directive (GCP; Directive 2005/28/EC) and a selection of ISO standards were considered in the development^16,17^. The hardware component of the wearable sensors is a Conformitè Europëenne (CE)-marked Class I Medical Device. It has been developed under the ISO13485 Quality Management System. Through the Qualification Opinion, the EMA endorsed SV95C as a secondary endpoint for ambulant Duchenne muscular dystrophy (DMD) trial participants aged over 5 years when measured with a valid and suitable wearable device^18^. The Qualification Opinion is not specific to ActiMyo, but hardware and software performance are included in the Opinion. This approach is common practice in EMA qualifications, as they aim at qualifying the approach or endpoint and not to endorse a specific device.

The application to the EMA was prepared by a multidisciplinary group of experts (paediatric neurologists, biostatisticians, physiotherapists, engineers, regulatory affairs specialists and patient representatives). A Letter of Intent for request of qualification was submitted in June 2017 and the final Qualification Opinion was adopted by the CHMP in April 2019^18^.

The qualification application was based on several global natural history and pivotal trials^18^ (summarised in Box 1). This was the first qualification procedure conducted by the EMA for a digital endpoint, so there were limited precedents to inform the specific requirements. The continuous dialogue throughout the process with the EMA was extremely helpful to come to an understanding of the evidentiary requirements and data interpretation. The absence of an approved medicinal product in DMD made the qualification of SV95C as a primary endpoint difficult because longitudinal data including demonstration of sensitivity to treatment effect would be needed. In this case, it was accepted that sensitivity to change could be illustrated by response to steroids, which are routinely used^18^.

Box 1. Data provided to support the qualification of the SV95C measure The SV95C application demonstrated how participants’ ambulation could be reliably measured, and included the following evidence:

- The duration of recording (optimal 180 h, minimal 50 h) was calculated to obtain a minimal variability and maximum sensitivity of the endpoint at baseline^18^
- The external validity was demonstrated through a moderate correlation at baseline with three measures that have been used as primary endpoints in previous pivotal trials, namely the Six-Minute Walking Test (6MWT)^32^, the North Star Ambulatory Assessment^33^ and the Four-Stair Climb^34^. The 95th percentile was selected to ensure the best standardised response mean^18^
- There was a need to understand the factors that could potentially influence the variability of the measure, such as patients’ compliance to wearing the device during recording periods, duration of recording periods, and time of the day and days of the week during which the measure is performed. It appeared that only the days of the week during which the measure is recorded could influence it slightly because trial participants walk more slowly during the weekend, and a plan was thus proposed to mitigate the risk of influence^18^
- Definition of the minimal clinically important difference (MCID) based on a population distribution, and an analogy with the 6MWT (for which MCID was defined the same way^18^)
- A set of age- and sex-matched controls and a plan for longitudinal follow-up of controls^18^
- The feasibility of collection of such an endpoint in a global study, with data on trial participants’ compliance^18^

### Challenges

A follow-on Qualification Opinion application has been submitted to the EMA to upgrade the use of SV95C as a primary efficacy endpoint and generalise the application of SV95C to other neuromuscular diseases characterised by a proximal muscle weakness leading to progressive difficulties in ambulation. Notably, for the application to be generalisable, the clinical meaningfulness of SV95C for any other indications should be adequately established. With traditional assessments used in clinical practice, relevant concepts of interests are being measured across multiple indications. As an example, the 6MWT is used to measure distance walked in 6 min in DMD and also in MS, Parkinson’s disease and chronic obstructive pulmonary disease^19–21^. It is important to determine a clinically meaningful threshold for the measure to be qualified in different contexts of use or indications. This threshold might differ across indications, but the concept of distance walked and how it relates to ambulation has relevance across those conditions. Many digital COAs, such as SV95C, currently used in clinical trials are not available in clinical practice. Leveraging these endpoints as primary endpoints in confirmatory clinical trials, even if they have been qualified from a regulatory standpoint, could be a challenge for sponsors since recognition as a standard of care measure by a broad range of therapeutic area experts is essential to translate results in clinical practice. Clinical experience and consensus on measurement is essential to support the contextualisation of clinical trial data and medicine labelling information.

### Solutions

From a regulatory standpoint, a solution to bridge the evidence requirements of SV95C for use in other conditions would be to rely on the analytical and technical validation of SV95C (e.g., addressing parameters such as accuracy, precision, selectivity, sensitivity, reproducibility and stability) whilst evaluating the clinical validity, meaningfulness and utility of the endpoint in the new condition. Historically, however, assessments such as the 6MWT have been adopted empirically without formally qualifying their validity nor defining their context of use. Similarly, if wearable devices capable of capturing SV95C would be available to practitioners, the generalisability of SV95C could be additionally supported by real-world use and experience in conditions where rapid limb movement is a relevant concept of interest.

To support the broad recognition and use of digital COAs, industry co-authors would welcome the opportunity for a clinical network of therapeutic area experts, device experts and patients to be consulted together at an early stage. To date, the involvement of NBs during the qualification process is in part limited by the MDR, which prevents NBs from being consulted. A more collaborative approach would be beneficial in preparation of more complex products at the interface of medicines and medical devices. Whilst DHTTs used in clinical practice are not in the scope of the QoNM and hence are outside the EMA remit, industry authors believe that involving device bodies and other clinical experts earlier in the development and qualification process could support the transition of DHTTs used as clinical trial endpoints to the collection of real-world evidence in clinical care settings post-approval. The evidentiary package and device status would need to be tailored accordingly to accommodate the real-world clinical use.

## Case study 2—Multidimensional digital endpoints to assess neurological function via smartphone sensor-based technology in a real-world environment in multiple sclerosis

### What is the technology/how is it used?

Two biotechnology/pharmaceutical companies aiming at improving measures of MS disease progression have engaged into a pre-competitive agreement seeking to increase the chance of (regulatory) acceptance of digital endpoints derived from two different smartphone-based applications (apps), which share common concepts. Their objective is to characterise and quantify disability based on multiple active tests (e.g., Cognitive Test [information processing speed], Draw a Shape and Pinching Tests, Two-Minute Walk Test and U-Turn Test) and passive monitoring of functional performances in neurological domains of cognition, upper extremity function, gait, balance and overall mobility. Floodlight™ MS consists of several software components: including a smartphone app and five CE-marked medical device software (MDSW) components intended to provide an objective measurement of the function of people living with MS in between clinical visits^22,23^. Konectom™ is also a smartphone-based, CE-marked MDSW with nine assessments, intended to be used as a performance-based and patient-reported outcome assessment tool to quantify neurological impairments (motor and cognitive functions) in people living with MS^24^.

The Floodlight MS and Konectom apps can be used as data collection tools to characterise treatment effects within clinical trials and as patient management tools in clinical practice to inform patient care. A parallel development approach could enable common disease measurements, hence, to be used not only during development of the medicinal product but also during patients’ treatment; it could generate better quality real-world datasets and potentially provide earlier treatment access for patients.

### Regulatory aspects

The Floodlight MS and Konectom apps are subject to the MDRs in Europe, currently classified as Class IIa MDSWs. In the context of using these apps in medicinal product clinical trials, they are subject to GCP and particularly Computer Software Validation (CSV)^25^. Digital endpoints derived from these smartphone apps could be subject to the qualification procedure. To explore this possibility, an ITF meeting was held with the EMA to initiate discussions on Floodlight MS and further Qualification Advice could follow for Floodlight MS/Konectom-derived digital endpoints.

### Challenges

**Fig. 3:**
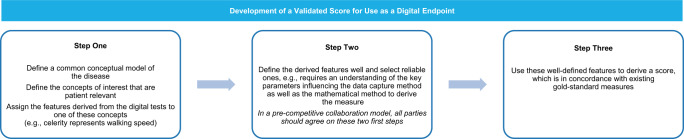
Summary of steps involved in the development of a validated digital endpoint. Three steps to develop a validated score for use as a digital endpoint.

Digital endpoint development is complex and includes several important steps leading to a validated disease measurement score (Fig. 3). The EMA qualification procedure mainly focuses on the first and last step in this process and proposes to address technicalities of algorithm development by providing information regarding CE-marked hardware specifications in the Qualification Opinion^11^. This approach does not examine how the science behind feature derivation would be addressed (e.g., software requirements, including pre-processing steps enabling raw data denoising, normalisation and segmentation) and which environmental factors may influence the results, which is a critical part of digital endpoint development. When assessing new medicinal products, the EMA needs reassurance that the algorithms used to derive endpoint results, and their subsequent modifications, perform as intended and are in line with the published Qualification Opinion to collect and interpret the data. At the time of MAA, the EMA requires that the company provides the relevant confirmation of software equivalence with the software used during the qualification from NBs or medical device NCAs (which only the developer of the method could access) or adequate bridging data to demonstrate comparability (which would be very difficult to generate without the software code). For analogue endpoints, the input data are reproducible in most cases and bridging for comparability is also achievable. In the case of a digital endpoint looking at the concept of celerity, for example (Fig. 3), to derive the feature ‘celerity’ captured from a test consisting of walking 2 min in a straight line, a sponsor would need to know several mathematical parameters used to calibrate the test, to normalise the data avoiding noise and understand what parameters drive variability in order to ensure the test performed is equivalent to the originator. To then demonstrate it is equivalent, several patients would need to be tested in a gait lab showing that the same input data (walking in a straight line for 2 min) provide the same output (e.g., the measure of celerity) across the two apps. Without the input and output data of the originator or the code behind the measure, this equivalent result is very difficult to achieve. Confirming equivalence of two different apps to derive endpoints is therefore challenging without having access to the software or input and output datasets or the software code. This leads to applicants relying on the developer of the DHTT in order to use qualified digital endpoints in their trials. Patients, healthcare professionals (HCPs), sponsors and regulators would benefit from having a harmonised set of easily accessible digital endpoints to drive adoption and support an easier contextualisation of treatment outcomes in the future, with fewer parallel development and no duplication of efforts.

Pre-competitive frameworks can support progression and consensus on digital measures; however, they are challenged by proprietary considerations and the ease with which technologies could be appropriated if the details on input requirements and systems design were to be disclosed.

### Solutions

For Floodlight MS, the sponsor held early interactions with the EMA ITF. Representatives of NCAs overseeing clinical investigations for medical devices participated in this discussion, along with two sponsor-invited NB representatives who attended in the role of observers to enable an informal exchange. So far, this meeting is the only example of interaction concomitantly involving the sponsor, EMA, NCAs and NBs. As per MDRs, NBs were limited in their ability to consult, hence setting up the meeting took several months. It was only possible to engage the NB because the company had an active open contract with the NB for other CE-marked products; this is not always the case for pharmaceutical sponsors starting their device development. NB participation brought expertise on how to approach components of software validation whilst NCAs informed on particularities of clinical investigation. One of the questions raised by the sponsor was how to claim equivalence of previous versions of the software generating the endpoint to newer versions. In this discussion, the advice of medical device regulators (NBs in particular) was very beneficial, considering the degree of algorithm changes and suggesting leveraging the Medical Devices Directive (MEDDEV) 2.7/1 guidance and MDR Annex XIV 3 on equivalence^26,27^. The medical device regulators from NCAs on the other hand, were able to advise whether the version of the software included in clinical trials qualified in that context of use as a medical device, which was essential information for the set-up of the study.

The QoNM has been a very helpful tool to support harmonisation of methods in medicinal product development. It has fostered the use of common methodologies for disease measurement, including endpoints used in clinical trials. Once a method is disclosed, in most instances, it can be used with limited reliance on the original developer of the method. With digital endpoints, demonstration of comparability of DHTTs is difficult and limits the ability of sponsors to use DHTTs without the collaboration of the technology developer who owns the intellectual property of the code. In addition, the level of information needed to bridge one software-processing method to another is too complex to be included in a document, so it is now done within the Qualification Opinion for traditional analogue endpoints. This static approach would also fail to address fast-evolving technology. The industry authors would see a repository of qualified software codes and associated methods to derive digital endpoints as a possible solution to guarantee the equivalence of endpoints across registrational studies. Having an independent third-party organisation governing such a repository could facilitate that qualification standards are met before submission of the request for a Qualification Opinion. Furthermore, by establishing a licensing model, industry and academia partners would be incentivised to publish their software on this platform for use by various sponsors, attesting to a level of quality.

The industry authors are of the opinion that experts from device bodies should be involved in the qualification procedure to advise sponsors on the validity of pre-processing steps and quality assessment procedures for feature processing (step 2 in Fig. 3). Subsequently, the same device experts could advise other sponsors on the equivalence of their technology to the qualified one. Such an approach would facilitate harmonisation of measurements, comparability of generated datasets, and could help drive ubiquity of use for digital endpoints.

## Case study 3—Digital diabetes management systems: connected insulin pen

### What is the technology/how is it used?

Several connected care systems are being developed to support treatment and management of diabetes^28^. Here we present a connected care system comprised of an insulin pen that connects to a smartphone app to automatically track insulin doses injected by patients.

Many patients struggle to manage their diabetes effectively because of the complexities associated with the treatment. Not only does insulin therapy require many steps and decisions, but also depends on manual recording of glucose and insulin data, which is burdensome and has poor patient compliance^28^. Automating the recording of blood sugar, insulin dose measurement and time of injection could not only ease the burden of manual recording, but may improve the accuracy of patient data. A more reliable and complete dataset has the potential to improve treatment management and outcome, which in turn could positively impact diabetes self-management.

### Regulatory aspects

A medical device used in combination with a medicinal product may have an impact on the benefit–risk of the product, for example it may decrease (or increase, if malfunctioning) the risk of medication errors. Therefore, the available regulatory pathway for a digitally based diabetes management system differs depending on the type of drug–device combination. The connected pen might be provided as a pre-filled or a reusable injector pen, and the connected component may be integral to the pen or an add-on device. These are important considerations, as the pre-filled pen containing insulin is authorised as a medicinal product (MDR Article 117 for Medicine–Device Combinations Regulation [EU] 2017/745); however, it will require a NB Opinion for the approval of the MAA (MDR Regulation [EU] 2017/745), whilst the reusable injector pen to be used with an insulin cartridge is authorised separately as a medical device^16^. Depending on the intended use, an app compatible for use with a connected autoinjector pen may have some functionalities that require the app to be certified as Class IIa or IIb MDSW. Additionally, the add-on device to a pre-filled pen or reusable pen may need to be certified as a medical device. The conformity assessment procedure for a MDSW is dependent on the risk class and may require the involvement of a NB^29^.

### Challenges

**Fig. 4:**
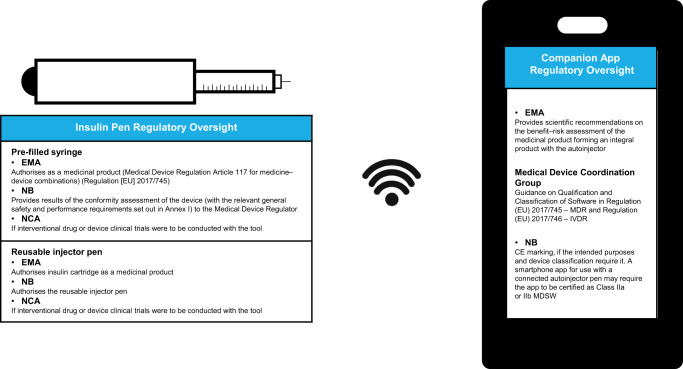
Regulatory considerations for an insulin pen-connected medical device. The roles and responsibilities of regulatory bodies in the assessment of medical devices used in combination with a medicinal product. *CE* Conformitè Europëenne, *EMA* European Medicines Agency, *EU* European Union, *IVDR* In Vitro Diagnostic Devices Regulation, *MDR* Medical Device Regulation, *MDSW* medical device software, *NB* Notified Body, *NCA* National Competent Authority.

The introduction of ‘connected’ devices raises challenges for sponsors on the nature and level of data necessary to support the MAA of the associated medicinal product. The evidence in relation to MDR compliance further varies depending on the classification of the DHTTs and whether integral, co-packed or supplied separately. The roles and responsibilities for the assessment of such medicinal product–medical device combinations (Fig. 4) can also fall in a grey area.

The EMA’s responsibility is to provide a scientific opinion on the benefit–risk assessment of the medicinal product of which the autoinjector pen is an integral part. With the implementation of the MDR, and specifically with MDR Article 117, in the review of the ‘connected system’, there may be some areas of overlap between the scope of the medical device regulatory framework (i.e., conformity with relevant General Safety and Performance Requirements) and the medicinal product benefit–risk assessment. The safety and performance of the medical device part could affect the benefit–risk of the medicinal product. The scope of the EMA review will consider the impact of the digital health application on the benefit–risk assessment of the medicinal product (e.g., accuracy of dose administration recording), as well as the approach taken by the applicant to evaluate and manage its impact, for instance, in the case of medication errors. Sponsors can seek CHMP input on data needed to support the MAA through the scientific advice procedure, which is well established^7^. However, the major challenge that sponsors face is navigating two regulatory frameworks simultaneously, which may have areas of overlap. To facilitate the approvability of these DHTTs, it is beneficial to have a clear understanding of the type of data required, clinical and other, to support any claims made in the respective medicinal product information and/or medical device instructions for use.

### Solutions

To overcome the current challenges and streamline the qualification and approvals of such DHTTs, sponsors would benefit from concurrent and aligned joint scientific advice between the medicinal product regulators and the medical device bodies. This is in line with the development of an integrated evaluation pathway for the assessment of medical devices and medicines, as mentioned in the EMA RSS to 2025^9^; its creation would facilitate timely alignment on advice and assessment of the different DHTTs components (i.e., medicinal product, medical devices and app).

## Case study 4—Digital monitoring of symptoms in patients with cancer

### What is the technology/how is it used?

Web-based digital patient monitoring (DPM) software modules are in development to capture symptoms reported by patients with cancer. This concept is an evolution of more traditional (telephone-based) remote patient monitoring. DPM is a tool to collect patient-reported outcomes in clinical practice and enable review by clinicians in real time. Modules are the patient-facing element of the software, which encompass a symptom questionnaire and in some cases algorithms for processing of the symptom questionnaire input and educational materials. When software modules are linked to a clinic’s electronic medical records or used with a standalone platform, the combination is described as a DPM solution. The patient-facing area of the DPM module generally focuses on a particular cancer and may contain or capture medicine-specific information. The aim of this technology is to improve patient care and healthcare resource utilisation by better managing the disease and medicinal treatment, through capturing patient-reported disease or treatment-related symptoms and quality-of-life-reported data, and facilitating seamless non-urgent communication between patients and HCPs.

### Regulatory aspects

DPM modules can improve compliance by enhancing the detection of adverse events directly reported by the patient, which may indirectly lead to improving the safety profile of a medicinal product as well as to improved clinical decision-making. In Europe, they are generally considered Class IIa MDSW apps (depending on the actual intended use) and are subject to conformity assessment (MDR)^16^. As modules have different intended applications and classifications, it may be necessary to evaluate each component of the DPM software to determine its medical device classification. When it is claimed that the DPM module has an impact on the benefit–risk of its associated medicinal product, it is important to be able to distinguish the contribution to the benefit–risk of each individual component.

### Challenges

DPM modules can inform clinical decision-making related to one medicinal product within a particular cancer type. The evidence required to extend the claim of the DPM module from its functionality on one medicinal product across a class of medicinal products, treatment lines or disease indications, is not clear. To evaluate the equivalence of evidence generated in one indication to another, or across medicinal products, a critical understanding of the medicinal product’s efficacy and safety features as well as specific disease area expertise are required. NBs responsible for the CE certification do have clinical experts, but they might not be familiar with the details of a medicinal product’s safety, efficacy and class effects. For products regulated as medical devices, such as DPM modules, it is generally not possible to engage formally with the EMA, unless there is scope for a claim in the medicinal product labelling. Hence, a challenge sponsors of medical devices face, within the current regulatory framework, is the complexity of involving the NBs, and potentially the EMA, in consultation on such queries.

In addition, several DPM modules developed by manufacturers are being brought to market claiming comparable functionality without being required to demonstrate comparability in treatment outcomes. Requiring evidence to demonstrate comparability of treatment outcomes across tools would be disproportionate for a medical device presenting a low risk to patients. Manufacturers of DPM modules aim to demonstrate that their DPM modules are safe to use, and the technical performance is adequate for their intended use. However, evidence of comparable functionality could better inform patients’ and physicians’ preferences for evidence-based disease management solutions.

### Solutions

Although DPM module regulation falls outside the EMA’s remit, the industry authors would welcome the opportunity of a joint discussion bringing together knowledge and expertise available across the NBs and the EMA. Overarching guidance on the evidence required to extend the claim of the DPM module from its functionality on one medicinal product across a class of medicinal products, treatment lines or disease indications, would be beneficial to sponsors. Additionally, with an increase in similar DPM modules being brought to market and claiming comparable functionality, it would be beneficial to developers if NBs defined the general specifications needed to demonstrate equivalence in achieving comparable treatment outcome for this type of DHTT.

## Discussion

This review aims to illustrate challenges of regulating digital health technologies that often sit at the intersection of two European regulatory frameworks (medical device and the medicinal product regulations).

As shown in the four case studies (CSs 1–4), summarised in Table 1, DHTTs have the potential to benefit medicines development and patient care; however, the complexity of the European regulatory framework and current unavailability of a pathway that allows for formal joint or parallel advice from medicine regulators and device bodies, may result in a slower uptake and development of DHTTs.

**Table 1:** Summary of case studies.

Case study	Type of DHTT	MDSW (yes/no)	Regulatory pathway	Proposed context of use of the Qualification Opinion	Disease area
1. Stride Velocity 95th Centile	Wearable sensors for passive measurement of disease phenotype	No	QoNM for Medicines Development	Use as a secondary endpoint in pivotal medicinal product studies, in ambulant Duchenne muscular dystrophy patients aged 5 years and above, when measured by a valid and suitable wearable device	Duchenne muscular dystrophy
			Device used to support qualification is a CE-marked hardware (ActiMyo®)		
2. Floodlight™ MS/Konectom™	Smartphone apps for active and passive measurement of disease phenotype	Floodlight MS tests Yes	QoNM for Medicines Development	Digital measures that detect subtle changes in individual MS disease course trajectories and enable the development of more sensitive, responsive and patient-relevant endpoints to complement or replace existing measures of disease progression in people living with MS	MS
			The Floodlight MS application presents assessments that are CE-marked MDSW for use in clinical practice. Various configurations of the Floodlight MS suite of tests are used in clinical trials within data collection DHTTs. DHTTs used in medicinal product clinical studies to support data collection only do not require a CE mark. They should be GCP compliant		
		Konectom Yes	Konectom is similarly a medical device app intended to be used as a performance-based outcome and patient-reported outcome assessment tool to quantify neurological impairments (motor and cognitive functions) in people living with MS. Various configurations of the Konectom suite of tests are also used in clinical trials within data collection DHTTs		
3. Connected insulin pen in diabetes	A connected care system comprised of an insulin pen smart component and a smartphone app for passive dose monitoring	Yes^a^	Medicinal product (forming an integral product) or medical device if the pen and/or the smart component is reusable	Insulin pen that connects to a smartphone app allowing automated tracking of doses injected by patients	Diabetes
			CE-marked MDSW for the digital component on the connected app	Smartphone apps that digitally track insulin doses already exist on the market as MDSW for use in clinical practice. Some might also automate other manual processes, such as tracking blood glucose through a connection to a blood glucose monitor and calculation of the bolus	
4. Digital patient monitoring for cancer treatment	A web-based digital patient monitoring software capturing patient-reported symptoms	Yes	CE-marked MDSW	Indication- or medicine-specific modules to improve patient care and healthcare resource utilisation by enabling cancer patients and healthcare professionals to better manage the disease	Cancer

*CE* Conformitè Europëenne, *DHTT* digital health technology tool, *GCP* Good Clinical Practice, *MDSW* medical device software, *MS* multiple sclerosis, *QoNM* Qualification of Novel Methodology.

^a^Dependent on the intended use.

In 2020, the European Commission adopted the Pharmaceutical Strategy for Europe, which provides opportunities to adapt legislation to be future proof^30^. It highlights important aspects of supporting the development of medical devices for use in medicine development, such as the need for more collaboration within the regulatory networks. The revision of the legislation coming with the Pharmaceutical Strategy for Europe is an opportunity to address some of the challenges presented by DHTTs highlighted in the case studies detailed in this paper. Potential solutions, as put forward by the authors, are discussed below.

### Increased collaboration between regulatory bodies

The opportunity for enhanced collaboration between the various stakeholders within the regulatory system is evident in all the four cases. Whilst the EMA scientific advice procedure and the EMA QoNM are well established for medicinal products, there is no equivalent procedure for getting advice for the medical device components used with medicinal products. The opportunity for structured joint feedback from medicine regulators and device bodies on DHTT development would be welcomed by medicinal product sponsors and technology developers.

Opportunities to create direct interaction between the EMA and technical experts from NBs are not foreseen under the current EU legislation. The Floodlight MS case study (CS2) is an example of an ad-hoc joint interaction (EMA, NCAs, NBs), where the NBs were able to advise on what evidence would be needed to claim equivalence of software versions to derive a digital measure; this advice was very beneficial. This interaction is not a standard interaction, and the introduction of the MDR specifying that the NBs have no ability to consult has made these opportunities for interaction even more complex.

The joint advice of the EMA, NBs and device regulators would foster and create efficiencies in the development of DHTTs, and a number of use cases have been presented. For the development of DHTTs with potential future use in a clinical practice, joint advice would be helpful to support the transition from clinical trial endpoints to clinical practice (CS1). Guidance on the demonstration of software equivalence would be beneficial for bridging and claiming comparable performance to measure a qualified endpoint (CS2). The input of software validation experts could be valuable when data generated using a DHTT are submitted as confirmatory evidence of clinical studies to support a medicine’s benefit–risk assessment (CS1 and CS2). In the future, DHTTs intended to be used in conjunction with a medicinal product will become more complex and may have a significant impact on the benefit–risk of medicinal products (CS3 and CS4). In such cases, all stakeholders would benefit from an integrated pathway assessment of the DHTTs throughout the lifecycle of the product, encompassing the best clinical and technology experts in the EU.

In recognition of the changing European legislation and fast-evolving landscape of medicinal products and medical devices, including DHTTs, the EMA has proposed an integrated evaluation pathway for the assessment of medicines used in combination with medical devices in their RSS 2025. In the future, as foreseen by the MDR, expert panels on medical devices will also provide scientific advice to manufacturers on their clinical development strategy and proposals for clinical investigations for certain high-risk medical devices, namely Class III devices and Class IIb active devices intended to administer and/or remove medicinal product(s). Whilst limited to certain devices and not covering the broad range of DHTTs, this proposal will foster collaboration between parties as multiple parallel assessments remain a challenge for developers aiming to guarantee timely access to novel therapeutics or methodologies such as DHTTs in Europe.

### More collaboration between companies enabled through multi-stakeholder platforms for sponsors

Sponsors developing DHTTs aiming at capturing endpoints for medicines development would benefit from collaboration platforms supporting pre-competitive efforts and digital endpoint implementation in clinical trials. The qualification process provides an opportunity for a high degree of alignment in digital measurements within diseases and could facilitate a faster uptake of DHTTs overall. A hindrance to harmonisation and deployment in trials is the (understandable) reluctance of developers to divulge proprietary software codes. One solution could be that the qualification provides high-level information on algorithm use and its references for eventual use by other sponsors. Alternatively, these algorithms could be made available in a sponsor-agnostic data space handled by an independent third party, which could provide an interesting solution. Algorithms, software codes or derivation methods from which qualified endpoints are derived could be shared and licensed to sponsors for use in studies. Such a space could also support pre-competitive collaboration and drive convergence in digital measurements. Pre-competitive collaborations would enable alignment of the digital features considered and consensus on their computational principles when developing a score for specific indications. This would enable equivalence of digital measures and harmonise practices between developers of DHTTs. Initial proposals for multi-stakeholder platforms exist, such as one led by the Digital Medicine Society with their digital endpoint library^31^. Ultimately, digital endpoints used to support authorisation of medicinal products should be interpretable and comparable across treatments and be open for use by as many sponsors as possible.

### Generalisability of digital measures and tools across various indications

Qualification of digital methodologies increases the transparency on the requirements needed to support the use of high-quality digital solutions for the benefit of patients and HCPs. This, in turn, enables device developers to adhere to quality principles and specifications required to reliably reproduce a clinical outcome generated with an equivalent tool.

A common question faced by sponsors is how to bridge the evidentiary requirements to support the qualification of an already qualified DHTT for use in other indications. With more experience gained on DHTTs, the industry authors would welcome EMA guidance on what evidence is necessary to support patient relevance of digital endpoints from one to another context of use and what evidence remains valid across contexts of use. Such guidance would provide important insights and foster the development of DHTTs in multiple conditions. Improving the generalisability of digital measures and tools across various indications is important to keep pace with the increasing rate of innovation in healthcare.

## Conclusion

The case studies presented outline the different regulatory frameworks that span across DHTTs used in medicines development and highlight the need for increased collaboration as well as potential evolution of the current regulatory frameworks to foster this. Enhancing collaboration between stakeholders and engaging the breadth of expertise available in the EU through multi-stakeholder assessments have the potential to expedite the development and qualification of novel DHTTs for use in medicinal product development. The use of DHTTs is expected to continue to increase in the future; harmonisation and increased collaboration across the different regulatory bodies and between sponsors, technology developers, HCPs and patients will be critical to ensure that medicinal product development can benefit from the fast-paced advances of technologies. A formal strategy to improve the integration of the assessment of medicines and medical devices when needed, would be welcomed by developers for predictability, consistency and efficiency. The upcoming Pharmaceutical Strategy for Europe is an important and timely opportunity for Europe and supports the need to address digital transformation in the EU, including how to address the implementation of ‘personalised medicine’ and highlighting the importance of collaboration and regulatory expertise sharing between medicine and device regulatory bodies^30^. All the key decision-makers in the healthcare ecosystem are being brought together to create tangible actions and new solutions for our healthcare systems. The EU has the building blocks to solve these challenges and seize the opportunities offered by innovative healthcare solutions to create better and sustainable healthcare for generations to come.

## Disclaimer

The views expressed in this article are the personal views of the author(s) and may not be understood or quoted as being made on behalf of or reflecting the position of the European Medicines Agency or one of its committees or working parties.

## Data Availability

The findings and data supporting this Review are derived from the authors’ insights and references cited within the paper.
